# Chiari I Malformation and Idiopathic Growth Hormone Deficiency Case Series

**DOI:** 10.7759/cureus.90868

**Published:** 2025-08-24

**Authors:** Nikolitsa Techlemetzi, Sokratis Katsoudas, Evangelia Tsitsekli, Ioannis Pichlinski, Ioulia Polychroni, Paraskevi Zosi

**Affiliations:** 1 Pediatrics, General Hospital of Nikaia Peiraia Agios Panteleimon, Nikaia, GRC; 2 Pediatric Endocrinology, Private Unit, Athens, GRC; 3 Pediatric Endocrinology, General Hospital of Nikaia Peiraia Agios Panteleimon, Nikaia, GRC

**Keywords:** chiari i malformation, gh, gh therapy, growth hormone deficiency, pediatrics, recombinant human growth hormone treatment, short stature, weekly gh therapy

## Abstract

Chiari I malformation (CIM) and idiopathic growth hormone deficiency (GHD) are distinct, yet occasionally coexisting conditions in pediatric patients. The underlying pathophysiological link between the two entities remains controversial. In this case series, we present five pediatric patients with concurrent CIM and idiopathic GHD, detailing their clinical presentations, diagnostic workups, and responses to recombinant human growth hormone (rhGH) therapy. Notably, three of the cases were asymptomatic for CIM, with the malformation incidentally detected during evaluations for short stature. All patients demonstrated a positive growth response to rhGH treatment, with no evidence of CIM progression over the monitoring period. Our literature review highlights several proposed mechanisms linking CIM and GHD, including the potential effects of breech delivery, congenital midline developmental anomalies, and an underdeveloped posterior cranial fossa. Despite concerns in some reports about the possible exacerbation of CIM symptoms with rhGH therapy, such as persistent headaches, cerebellar ataxia, or spinal cord-related symptoms, our findings suggest that, when administered under careful multidisciplinary supervision, rhGH can effectively promote growth without adversely affecting CIM. To conclude, we highlight the importance of close collaboration between endocrinologists and neurologists in managing patients with these coexisting conditions.

## Introduction

Idiopathic growth hormone deficiency (GHD) is a broad term that includes some children with distinctive pathophysiology that may be presented radiologically and others in whom the pathological insult is unknown and the explanation for the GHD is unclear [1]. In pediatric patients, isolated idiopathic GHD is the most common cause of GHD [2]. GHD is typically characterized by short stature and a low growth velocity relative to age and pubertal stage. The variability and age of presentation largely depend on the onset and severity of GHD, with growth velocity impairment correlating with its severity [3]. Those with complete growth hormone (GH) deficiency due to a GH gene deletion exhibit the most pronounced effects. Alternative causes of impaired growth should be evaluated and ruled out, and the diagnosis is confirmed via GH stimulation tests [1].

Chiari malformations were first described in the 1890s by Dr. Hans Chiari, professor of pathological anatomy at the German University in Prague, who found in cadavers four congenital anomalies of the brain [4]. These malformations were later named Chiari malformations (I-IV), and all four include the hindbrain. The first three consist of different degrees of hindbrain herniation, and the fourth consists of cerebellar hypoplasia or aplasia. While the Chiari classification is useful for categorizing the majority of patients, it is now believed that the malformations are distinct pathophysiological and anatomical entities [5].

Chiari I malformation (CIM) is the most common of all four types, with the prevalence reported to be 0.24-3.6% in the general population, with greater estimates observed in younger ages [6]. It describes the condition in which the cerebellar tonsils descend more than 3-5 mm below the foramen magnum into the spinal canal [5] and is frequently associated with syringomyelia due to the altered flow of the cerebrospinal fluid [7]. It can be congenital or secondary due to space-occupying lesions such as tumors or due to hydrocephalus [8]. The CIM can either be asymptomatic or present with a variety of symptoms, including symptoms from the cerebellum, the brainstem, or the cervical cord [5].

The prevalence of CIM and idiopathic GHD has been reported at approximately 4% [6]. The association between GHD and CIM, however, remains debated. Over the years, various mechanisms have been proposed, along with concerns regarding the potential impact of recombinant human growth hormone (rhGH) therapy on malformation progression. Furthermore, our literature review identified a few documented cases of CIM with idiopathic GHD, and none to our knowledge describing patients treated with weekly rhGH therapy, as in one of the five cases presented here. This series, therefore, provides a valuable contribution to the existing literature.

## Case presentation

Case 1

A nine-year-old girl was referred to a private practice due to short stature. She was born via full-term normal delivery at 39 weeks of gestation without any trauma, with a birth weight of 2,510 g (-1.9 SDS) and a length of 48 cm (-0.6 SDS). From her medical history, she is under treatment for hypothyroidism and has CIM. The CIM was diagnosed at three years of age due to vomiting via magnetic resonance imaging (MRI), which showed prolapse of cerebellar tonsils and development of syringomyelia in the cervical spine, and due to the development of dysphagia and frequent mild headaches at four years of age, she underwent decompressive surgery. Since then, she has been asymptomatic.

**Figure 1:**
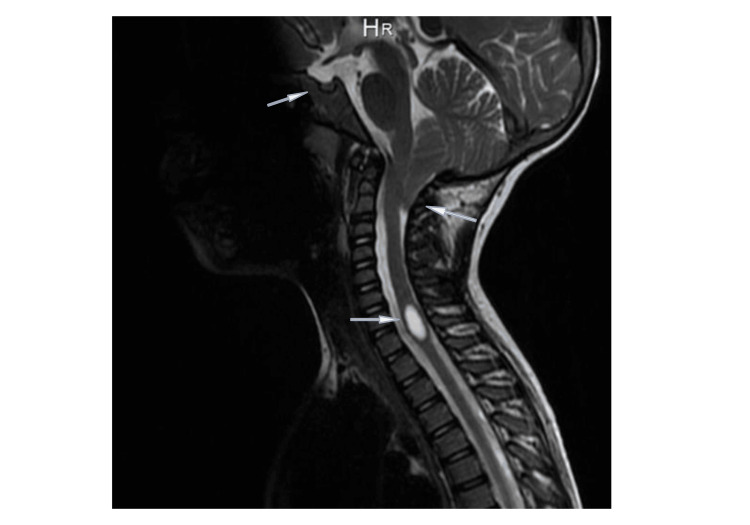
MRI of the first patient before the initiation of GH therapy, which presents a slight prolapse of the cerebellar tonsils, syringomyelia in the cervical spine, and a pituitary with slightly smaller measurements than normal. MRI: Magnetic Resonance Imaging, GH: Growth Hormone

**Figure 2:**
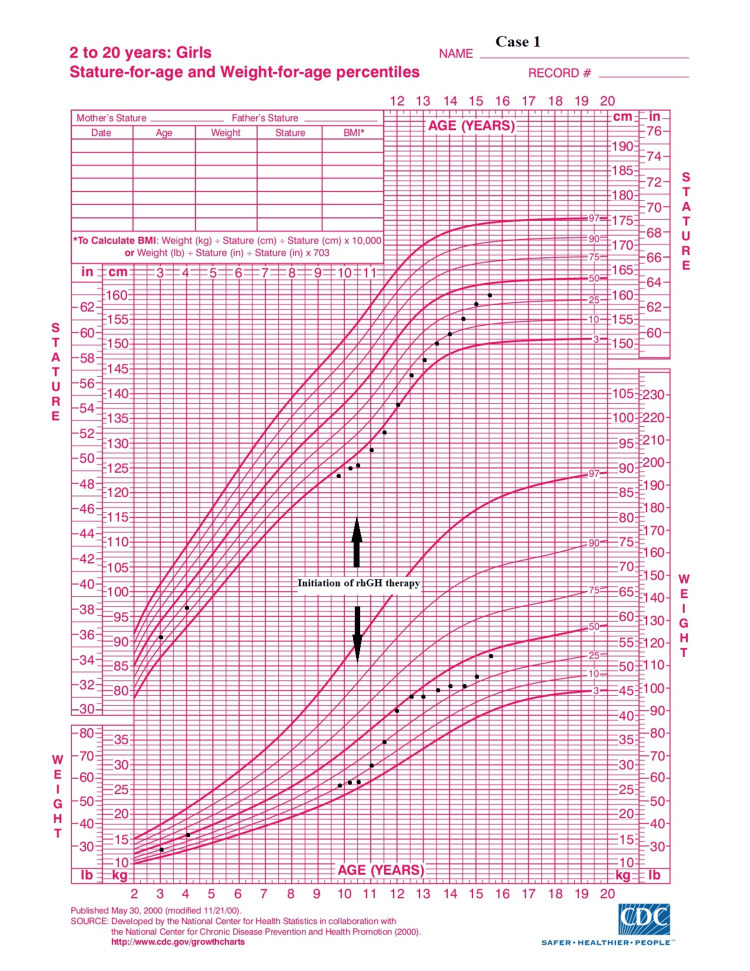
Growth chart: case 1 Adapted from Centers for Disease Control and Prevention growth chart

At nine years and eight months of age, she had a weight of 26 kg (-1.12 SDS), a height of 123.5 cm (-2.06 SDS), a height velocity of 3.5 cm per year (-2.45 SDS), and a bone age of eight years and six months. Mid-parental height was calculated to be 153 cm ± 8.5 cm. Tanner stage for pubic hair was stage II, and for breast development, stage I. Laboratory workup revealed a normal complete blood count and blood chemistry tests. Autoimmune antibody screening showed that total IgA and tTG-IgA antibodies were all within normal limits, excluding the diagnosis of celiac disease. Hormonal levels were also normal. MRI of the pituitary, as part of screening, showed slight prolapse of the cerebellar tonsils and syringomyelia, which were the same findings as the MRI after the decompressive surgery, and a pituitary gland with slightly smaller measurements than normal (Figure 1). GH peak levels in the insulin and L-DOPA tests were 6.49 ng/ml and 2.479 ng/ml, respectively; therefore, she was diagnosed with idiopathic GHD. Two applications for the administration of treatment with rhGH to the Central Board of Health, which is an advisory body related to the structure and operation of the National Health System in Greece and whose permission for initiating rhGH is mandatory, were rejected due to insufficient knowledge on the use of rhGH in patients with idiopathic GH deficiency and CIM. A new application was filed when the patient was 10 years and 6 months old with a weight of 26.5 kg (-1.60 SDS), a height of 125.5 cm (-2.28 SDS), a height velocity of 2.1 cm per year (-3.16 SDS), and a bone age of nine years and nine months. The Tanner stage for both pubic hair and breast development was stage II. The permission was given by the Central Board of Health; thus, the patient was initiated on treatment with daily rhGH at a dose of 0.1 mg/kg per week, divided into daily doses. She has been evaluated every six months and responded well to the treatment, as is shown in the growth chart (Figure 2). The patient has been in treatment with rhGH for five years and has not presented any new symptoms regarding the CIM. The last MRI, which was performed after four years of treatment, showed no alterations in comparison with the MRI performed before starting treatment.

Case 2

A six-year-old boy was referred to the private practice due to short stature. He was born via normal delivery at 35^+4^ weeks of gestation, with a birth weight of 3,150 g (+1.6 SDS) and a length of 52 cm (+2 SDS). More information about the gestation and perinatal period was not obtained because he was born in another country, and his parents could not provide a full history. From his medical history, he has CIM, which was diagnosed at five years of age due to vomiting via MRI, and he underwent decompressive surgery the same year. Since then, he has not shown any symptoms regarding the CIM. From the family history, he has a brother with idiopathic GHD.

**Figure 3:**
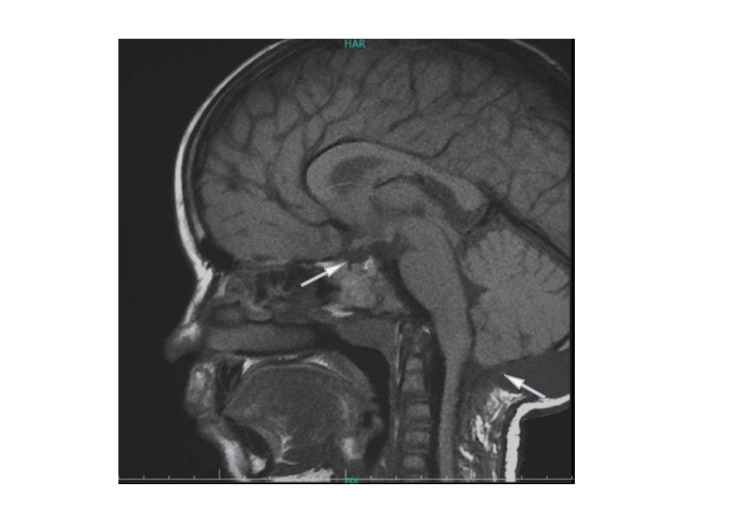
MRI of the second patient before the initiation of GH therapy, which presents slightly smaller measurements of the pituitary without any prolapse of the cerebellar tonsils. MRI: Magnetic Resonance Imaging, GH: Growth Hormone

**Figure 4:**
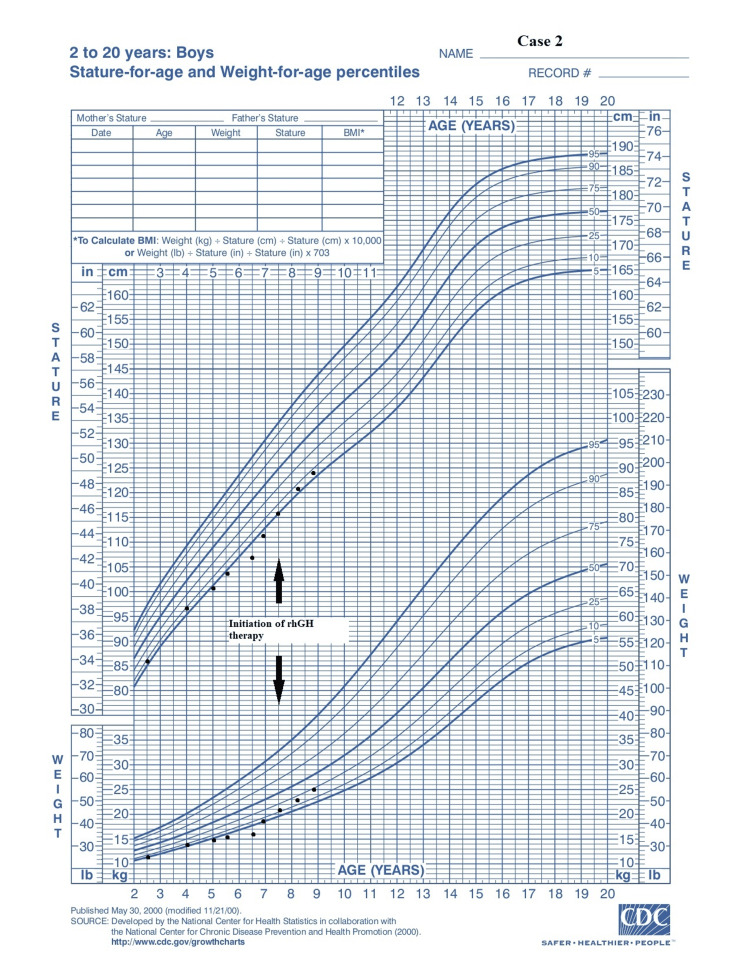
Growth chart: case 2 Adapted from Centers for Disease Control and Prevention growth chart

At six years and six months of age, he had a weight of 16.5 kg (-2.36 SDS), a height of 107 cm (-2.27 SDS), a height velocity of 2.4 cm per year (-3.88 SDS), and a bone age of five years and six months. Mid-parental height was calculated to be 170.5 cm ± 8.5 cm. Tanner stage for pubic hair was stage I, and his testicular volume was 3 ml. Laboratory workup revealed a normal complete blood count and blood chemistry tests. Autoimmune antibody screening showed that total IgA and tTG-IgA antibodies were all within normal limits, excluding the diagnosis of celiac disease. Hormonal levels were also normal. The MRI of the pituitary showed slightly smaller measurements of the pituitary without any prolapse of the cerebellar tonsils (Figure 3), which were the same findings as the MRI after the decompressive surgery. GH peak levels in the glucagon and L-DOPA tests were 3.3 ng/ml and 6.7 ng/ml, respectively; therefore, he was diagnosed with idiopathic GHD. He started daily rhGH therapy at a dose of 0.2 mg/kg per week, divided into daily doses, and continued for two years, during which he responded well to the treatment, as shown in the growth chart (Figure 4). The treatment was discontinued due to the family's relocation to another country. Throughout the treatment, he did not exhibit new CIM-related symptoms.

Case 3

**Figure 5:**
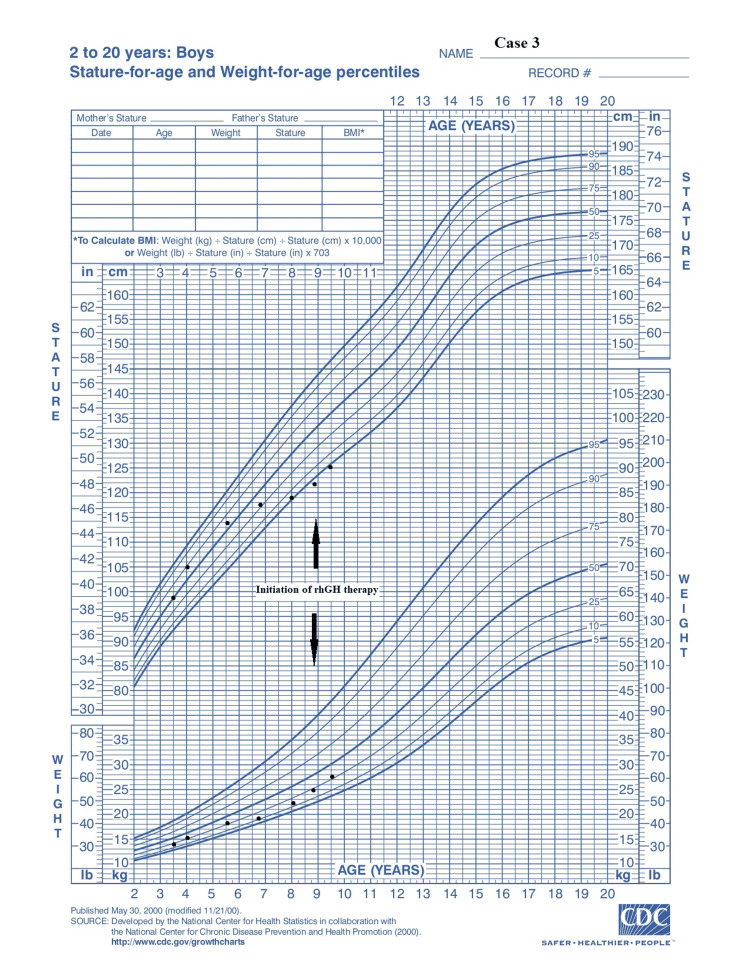
Growth chart: case 3 Adapted from Centers for Disease Control and Prevention growth chart

An eight-year-old boy was referred to the private practice due to short stature. The patient was delivered via caesarean section due to a prior caesarean delivery at 34^+2^ weeks of gestation. Birth weight was 2,630 g (+ 0.9 SDS), and length was 49 cm (+ 1.3 SDS). At eight years and ten months of age, he had a weight of 25 kg (-0.78 SDS), a height of 121.5 cm (-1.90 SDS), a height velocity of 2 cm per year (-4.31 SDS) for the last two years, and a bone age of six years and six months. His mid-parental height was calculated to be 179.5 cm ± 8.5 cm. Tanner stage for pubic hair was stage I, and his testicular volume was 3 ml. Laboratory workup revealed a normal complete blood count and blood chemistry tests. Autoimmune antibody screening showed that total IgA and tTG-IgA antibodies were all within normal limits, excluding the diagnosis of celiac disease. Hormonal levels were also normal. MRI of the pituitary showed slightly smaller measurements of the pituitary and the presence of CIM. The patient had no symptoms regarding the CIM. GH peak levels in the glucagon and L-DOPA tests were 4.2 ng/ml and 6.8 ng/ml, respectively; therefore, he was diagnosed with idiopathic GHD. He started treatment with weekly rhGH therapy at a single dose of 0.66 mg/kg per week. He has been on treatment for six months, in which he responded well to the therapy, as shown in the growth chart (Figure 5). Throughout his treatment, he remained asymptomatic regarding the CIM.

Case 4

**Figure 6:**
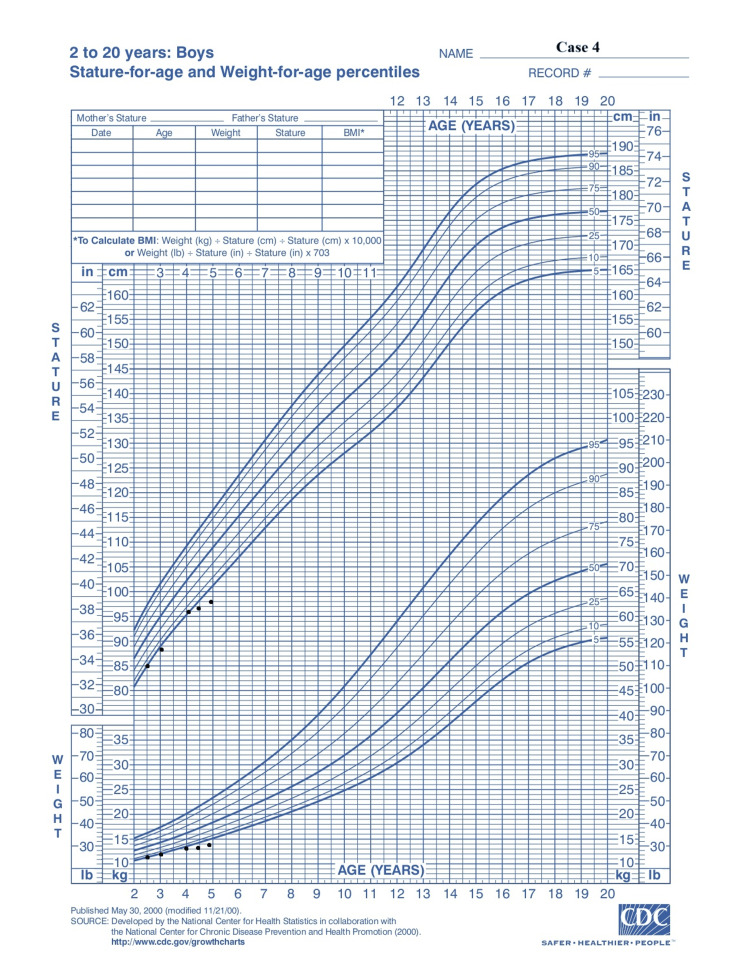
Growth chart: case 4 Adapted from Centers for Disease Control and Prevention growth chart

A five-year-old boy was referred to the private practice due to short stature. He was born by normal delivery at 38^+6^ weeks of gestation, with a birth weight of 2,760 g (-1.36 SDS) and a length of 51 cm (+0.5 SDS). At five years and two months of age, he had a weight of 13.5 kg (-3.03 SDS), a height of 99 cm (-3.08 SDS) (Figure 6), a height velocity of 2.5 cm per year (-3.96 SDS), and a bone age of three years. Mid-parental height was calculated to be 174.5 cm ± 8.5 cm. Tanner stage for pubic hair was stage I, and his testicular volume was 2 ml. Laboratory workup revealed a normal complete blood count and blood chemistry tests. Autoimmune antibody screening showed that total IgA and tTG-IgA antibodies were all within normal limits, excluding the diagnosis of celiac disease. Hormonal levels were also normal. MRI of the pituitary was normal except for the presence of CIM. The patient had no symptoms regarding the CIM. GH peak levels in the glucagon and L-DOPA tests were 4.4 ng/ml and 6.2 ng/ml, respectively; therefore, he was diagnosed with idiopathic GHD. He is to start treatment with daily rhGH therapy at a dose of 0.2 mg/kg per week, divided into daily doses, and the final outcomes remain pending.

Case 5

**Figure 7:**
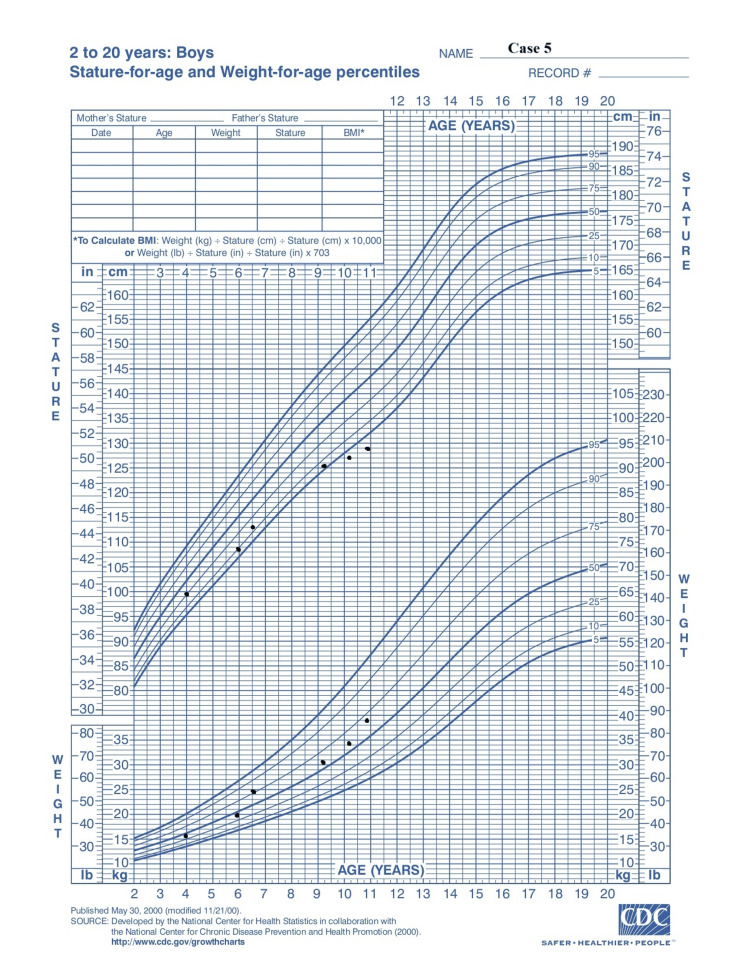
Growth chart: case 5 Adapted from Centers for Disease Control and Prevention growth chart

A 10-year-old boy was referred to the pediatric endocrinology department of the hospital due to short stature. He was born via caesarean section at 40 weeks of gestation, with a birth weight of 3,350 g (-0.10 SDS) and a length of 48.5 cm (-0.7 SDS). It was a twin pregnancy, but one twin was aborted during the sixth month. At 10 years and 10 months of age, he had a weight of 39.8 kg (+0.59 SDS), a height of 129 cm (-2.05 SDS) (Figure 7), a height velocity below 4 cm per year (<-3.02 SDS), and a bone age of six years and six months. His mid-parental height was calculated to be 175 cm ± 8.5 cm. Tanner stage for pubic hair was stage I, and his testicular volume was 2 ml. Laboratory workup revealed a normal complete blood count and blood chemistry tests. Autoimmune antibody screening showed that total IgA and tTG-IgA antibodies were all within normal limits, excluding the diagnosis of celiac disease. Hormonal levels were also normal. MRI of the pituitary was normal except for the presence of CIM. The patient had no symptoms regarding the CIM. GH peak levels in the glucagon and L-DOPA tests were 3.9 ng/ml and 2.9 ng/ml, respectively; therefore, he was diagnosed with idiopathic GHD. Daily rhGH therapy is to be commenced, and the final outcomes remain pending. Table 1 shows the overview of the cases presented.

**Table 1:** Overview of the cases presented rhGH: recombinant human growth hormone, CIM: Chiari I malformation, GH: growth hormone

Case Number	Age of initiation of rhGH treatment (years + months)	Sex	Presenting CIM symptoms	Prior CIM surgery	Peak GH level(ng/ml)	rhGH regimen	Duration of follow-up	CIM outcome on treatment
1	10+6	Female	No	Yes	6.49	Daily	4 years	No change in symptoms
2	6+6	Male	No	Yes	6.7	Daily	2 years	No change in symptoms
3	8+10	Male	No	No	6.8	Weekly	6 months	No change in symptoms
4	5+2	Male	No	No	6.2	Daily	treatment not yet initiated	Pending
5	10+10	Male	No	No	3.9	Daily	treatment not yet initiated	Pending

## Discussion

We described five cases of patients with CIM and idiopathic GH deficiency. The reported prevalence of CIM and idiopathic GHD is approximately 4% [6,9]. However, the relationship between GHD and CIM remains a topic of ongoing debate, with various theories proposed over the years. Fujita et al. proposed that the correlation between the two entities may result from injury during breech delivery. According to their theory, breech delivery can lead to idiopathic hypopituitarism due to ischemic or hemorrhagic damage to the pituitary gland. In addition, traction applied to the legs results in stretching of the spinal cord, which can cause pulling of the midbrain and cerebellum into the foramen magnum, creating the Chiari malformation [10]. Nevertheless, their theory cannot explain the incidence of CIM and idiopathic GHD in those who were delivered in a vertex position. Another theory suggested that the two conditions are grouped as congenital «midline» anomalies since both the cerebellar tonsils and the pituitary are developed in the fourth to eighth week of gestation, and mutations in genes involved in organogenesis, such as LHX4 and HESX1, have been linked to both conditions [11]. Last but not least, it is suggested that the undeveloped structure of the posterior cranial fossa in patients with GHD can lead to herniation of the brain stem due to the congestion in the posterior cranial fossa and result in CIM [12].

Notably, in three out of the five cases described, the patients had not presented any symptoms related to CIM, and the diagnosis was established by MRI in the context of investigating short stature. After reviewing the literature, there seems to be a high prevalence of incidental findings of CIM in patients with growth hormone abnormalities [13,14], which, in our opinion, highlights the critical need for thorough neurological evaluations during pediatric endocrine assessments.

In addition, the role of growth hormone in CIM remains a point of disagreement in the literature. In some cases, the initiation of rhGH therapy seems to worsen the symptoms of the CIM [10,15] or present symptoms, like central sleep apnea, in previously asymptomatic cases [8,16,17]. In these cases, the possible theories for the cause of this deterioration are the increase of intracranial pressure due to the potential action of GH and IGF-1 on the choroid plexus, which causes cerebrospinal fluid overproduction [6], and differential growth between the bony structures of the skull and the vertebral column. According to the latter, accelerated growth of the body leads to excessive stretching of the spinal cord and compression at the craniocervical junction [8]. In other cases, as well as in our described cases, the patient's symptoms remained unchanged [18,19,20]. Interestingly, two cases have been described in which, after the initiation of GH therapy, the CIM seemed to improve [21,22].

**Figure 8:**
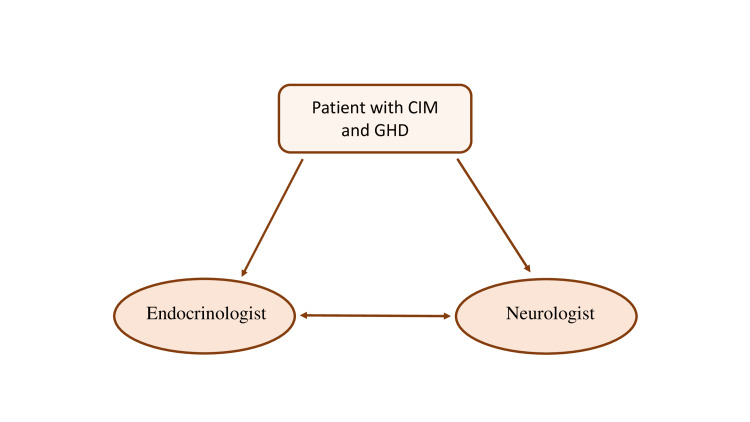
Multidisciplinary approach of a patient with CIM and GHD. CIM: Chiari I malformation, GHD: Growth hormone deficiency

Although we agree with the opinion of Ballard et al. that the GH therapy does not seem to interfere with the natural progression of CIM in the majority of patients and rhGH therapy should be prescribed when indicated, we emphasize that patients must be closely monitored throughout the course of therapy with annual assessments by a neurologist and MRIs if neurological symptoms arise. Long-term follow-up should involve both an endocrinologist and a neurologist, ensuring coordinated care through interdisciplinary collaboration (Figure 8). In their review to support this hypothesis, the authors present a data collection from 20 patients with CIM, some of whom had been on rhGH therapy and some who were not. Alterations in the MRI in both groups of patients were not considerably different [23].

In all three cases presented, there is a positive growth response to the treatment, which is apparent from their growth charts (Figures 2, 4-6, 7). After a literature review, we found no reported cases, to our knowledge, of patients with CIM receiving weekly rhGH therapy, making it difficult to assess treatment effectiveness or to compare its potential impact on CIM progression with that of daily administration. Therefore, we consider our contribution to case 3 to be significant in this regard. However, due to the short duration of the treatment period, there are limitations regarding the available data on the efficacy of rhGH therapy and its potential impact on the CIM.

## Conclusions

In conclusion, rhGH therapy in patients with CIM shows positive results in growth development. While treatment in the majority of patients does not seem to interfere with the natural progression of the malformation, we strongly recommend close cooperation between neurologists and endocrinologists to ensure appropriate follow-up. Moreover, the incidental detection of CIM in patients evaluated for short stature underscores the importance of comprehensive neurological assessment in pediatric endocrine workups. Finally, while our results are promising, they emphasize the need for further long-term studies involving larger cohorts to refine treatment protocols, elucidate underlying mechanisms, and ensure that growth promotion is achieved without adverse neurological effects.
